# Energy-Saving Synthesis of Functional CoS_2_/rGO Interlayer With Enhanced Conversion Kinetics for High-Performance Lithium-Sulfur Batteries

**DOI:** 10.3389/fchem.2021.830485

**Published:** 2022-02-10

**Authors:** Junan Feng, Yahui Li, Jinshi Yuan, Yuling Zhao, Jianmin Zhang, Fengyun Wang, Jie Tang, Jianjun Song

**Affiliations:** ^1^ College of Physics, Qingdao University, Qingdao, China; ^2^ National Engineering Research Center for Intelligent Electrical Vehicle Power System (Qingdao), College of Mechanical and Electrical Engineering, Qingdao University, Qingdao, China; ^3^ National Institute for Materials Science, Tsukuba, Japan

**Keywords:** cobalt disulfide, microwave hydrothermal, conversion kinetics, shuttle effect, lithium-sulfur battery

## Abstract

Lithium sulfur (Li-S) battery has exhibited great application potential in next-generation high-density secondary battery systems due to their excellent energy density and high specific capacity. However, the practical industrialization of Li-S battery is still affected by the low conductivity of sulfur and its discharge product (Li_2_S_2_/Li_2_S), the shuttle effect of lithium polysulfide (Li_2_S_n_, 4 ≤ n ≤ 8) during charging/discharging process and so on. Here, cobalt disulfide/reduced graphene oxide (CoS_2_/rGO) composites were easily and efficiently prepared through an energy-saving microwave-assisted hydrothermal method and employed as functional interlayer on commercial polypropylene separator to enhance the electrochemical performance of Li-S battery. As a physical barrier and second current collector, the porous conductive rGO can relieve the shuttle effect of polysulfides and ensure fast electron/ion transfer. Polar CoS_2_ nanoparticles uniformly distributed on rGO provide strong chemical adsorption to capture polysulfides. Benefitting from the synergy of physical and chemical constraints on polysulfides, the Li-S battery with CoS_2_/rGO functional separator exhibits enhanced conversion kinetics and excellent electrochemical performance with a high cycling initial capacity of 1,122.3 mAh g^−1^ at 0.2 C, good rate capabilities with 583.9 mAh g^−1^ at 2 C, and long-term cycle stability (decay rate of 0.08% per cycle at 0.5 C). This work provides an efficient and energy/time-saving microwave hydrothermal method for the synthesis of functional materials in stable Li-S battery.

## Introduction

Lithium-ion batteries systems have played a crucial role in the field of energy storage over the past two decades (Lu, et al., 2013; Manthiram, 2017; Ma, et al., 2021a). However, traditional lithium-ion battery electrode materials (LiCoO_2_, LiMn_2_O_4_, LiFePO_4_, etc.) cannot satisfy the requirement for high energy density in practical applications due to their limited energy density (Wang et al., 2015; Liu et al., 2020b; Zhang F. et al., 2021). With the development of energy technology in electronic devices and new energy vehicles, energy storage systems with low prices, that are environment friendly, and with excellent energy density have attracted great attention (Manthiram, et al., 2014; Bhargav, et al., 2020; Guo, et al., 2022). Lithium-sulfur (Li-S) battery has exhibited great application potential in next-generation high-density battery systems due to its high specific capacity (1,672 mAh g^−1^) and gratifying theoretical energy density (2,567 Wh kg^−1^) (Pang et al., 2016; Li Y. et al., 2018). However, the commercial viability of high-efficiency Li-S battery is limited by a series of shortcomings. Due to the low conductivity of sulfur and Li_2_S_2_/Li_2_S (Chung and Manthiram, 2018; Zhao Z. et al., 2020), the serious shuttle effect of polysulfides soluble in electrolyte (LiPSs) (Li_2_S_n_, 4 ≤ n ≤ 8) during charging/discharging, and inevitable growth of lithium dendrites (Zhou et al., 2021), the cycle stability of Li-S battery is unsatisfactory, which seriously hinders the development of Li-S battery (Xu et al., 2018; Hu et al., 2020). So far, numerous methods have been developed to solve these problems, including designing suitable cathode materials (Chen et al., 2017; You et al., 2019; Yan et al., 2020), modifying separators (Bai et al., 2016; Guo et al., 2019; Hu et al., 2021), and optimizing electrolytes (Agostini et al., 2015; Wang et al., 2016; Wan, et al., 2021). Among them, the simplest and most direct strategy is to modify the separator by constructing a reasonable functional interlayer to limit the severe shuttle effect (Rana et al., 2019; Wei et al., 2020).

As a key component of Li-S battery, the separator mainly prevents internal short circuits and provides a transmission path for ions (Ghazi et al., 2017). However, the conventional separator cannot suppress the shuttle effect of polysulfides owing to its highly micron-scale pore structure. In this case, coating a thin functional interlayer separator on the cathode side has proven to be a rational method, which can significantly immobilize polysulfides, improve the utilization of sulfur, and prevent the growth of lithium dendrite on the anode side (Li et al., 2017; Zhao et al., 2021). The functional separator facing the cathode electrode is the first barrier to limit the polysulfide, greatly increasing the utilization rate of sulfur species, and has attracted widespread attention in recent years (Zhang et al., 2015; Song et al., 2016; Liu et al., 2021). A variety of materials have been studied as functional interlayers for preventing the shuttle of polysulfides (LiPSs). First, one-dimensional (1D) (Chung and Manthiram, 2014; Gu, et al., 2020; Lin, et al., 2021) or two-dimensional (2D) (Wu et al., 2016; Lei et al., 2018; Zhang et al., 2018; Song et al., 2019) materials are introduced as the physical function coating to functionalize the conventional separator. Considering that polar materials can lead to chemical bonding with polysulfides, some polar transition metal compounds (CeO_2_, MnO_2_, CoS_2_, Co_9_S_8_, MoS_2_, Ni_2_P, etc.) were subsequently studied (Zhou et al., 2016; Li G. et al., 2018; Song et al., 2018; Tan et al., 2018; Li et al., 2020; Zhang H. et al., 2021), which can not only provide strong chemical interaction with soluble polysulfides but also play a role in electrochemical catalysis to promote redox reaction kinetics and reduce electrochemical polarization.

**SCHEME 1:**
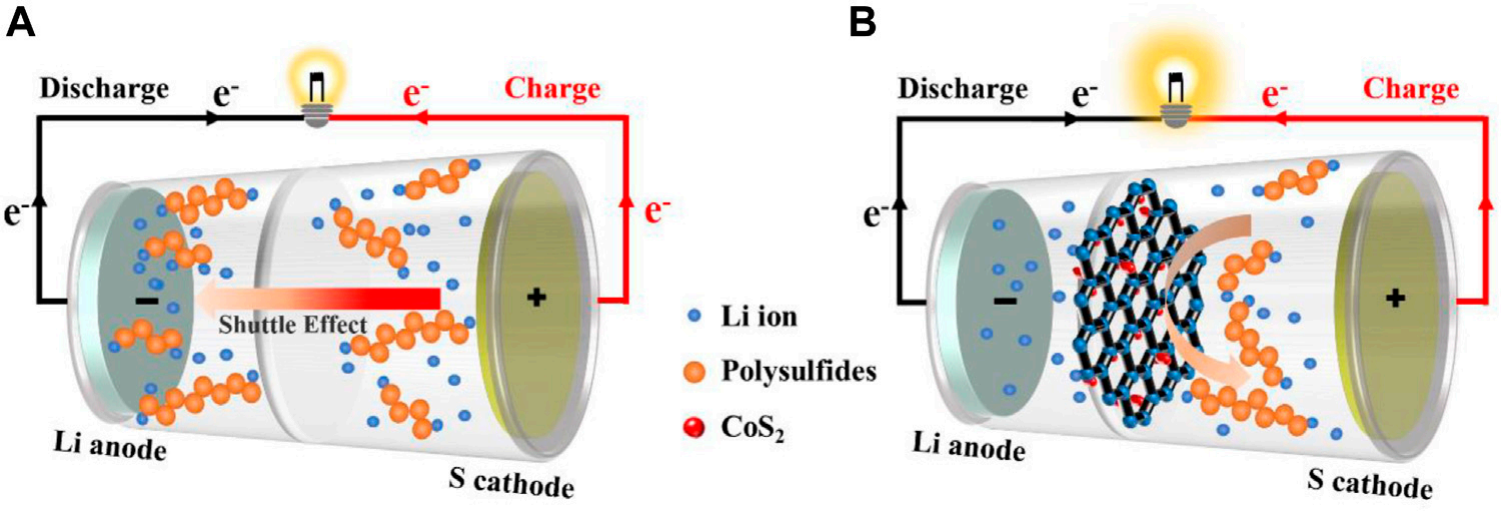
Schematic diagram of the cells without **(A)** and with **(B)** functional CoS_2_/rGO interlayer.

In this work, for the first time, we report an energy-saving synthesis of 3D porous CoS_2_/rGO composites as functional interlayer through an efficient microwave-assisted hydrothermal method to improve the electrochemical performance of Li-S battery. The microwave hydrothermal method has the advantages of fast nucleation speed, short reaction time, and energy conservation, and thus it is beneficial to improve the synthesis efficiency of composite materials (Kim et al., 2016; Yuan et al., 2016). The porous conductive graphene can serve as both the first barrier to physically block LiPSs and a second current collector to reduce electrochemical resistance, promoting electron/ion transfer (Zhang et al., 2020). Meanwhile, CoS_2_ particles which are *in situ* grown in the network of reduced graphene oxide (rGO) could not only improve adsorption ability for LiPSs but also act as catalytic centers to ensure the fast electrochemical redox conversion kinetics, further minimizing the loss of active materials (Abdul Razzaq et al., 2020). Therefore, as illustrated in Scheme 1, due to the double blocking of physical barrier and chemical interaction, compared with the traditional polypropylene (PP) separators, the functional CoS_2_/rGO modified separator effectively eases the shuttle effect of LiPSs, accelerates the redox reaction kinetics, and thus improves the cycle stability and rate performance of the Li-S battery.

## Experimental Section

### Preparation of CoS_2_/rGO Composite

Graphene oxide (GO) was synthesized by a modified Hummers method. Disperse 30 mg GO into 40 ml solution and sonicate for 1 h. Add 40 mg CoCl_2_·6H_2_O and 80 mg CH_4_N_2_S into the above dispersion and stir for 10 min. Then, pour it into a digestion tank and allow to react for 5 min under microwave hydrothermal process. After the reaction, the product was repeatedly washed several times and then freeze-dried for 24 h to obtain CoS_2_/rGO composites. In addition, the rGO was also synthesized according to the same route without the addition of CoCl_2_·6H_2_O and CH_4_N_2_S.

### Preparation of Cathode

Sulfur and carbon black (sulfur:carbon black = 7:3) are mixed and ground uniformly and then transferred to a polytetrafluoroethylene container for reaction at 155°C for 12 h to obtain sulfur and carbon black (S/C) composites. The S/C cathode slurry was prepared by uniformly mixing 80% S/C, 10% carbon black additive, and 10% polyvinylidene difluoride (PVDF) in *N*-methyl-2-pyrrolidone (NMP) and then was evenly coated on the aluminum foil and dried under vacuum at 60°C for 12 h. The dried aluminum foil electrode is cut into a disc with a diameter of 12 mm, and the S loading of the cathode is 1 mg cm^−2^.

### Preparation of CoS_2_/rGO or rGO Modified Separator

The CoS_2_/rGO composite or rGO prepared by the microwave hydrothermal method was mixed with PVDF with a mass ratio of 9:1 in NMP. The slurry was ground uniformly then coated on one side of the commercial PP separator and dried under vacuum at 60°C for 12 h. The modified separators are cut into 19 mm discs, and the mass loading of CoS_2_/rGO or rGO is only about 0.19 mg cm^−2^.

### Electrochemical Measurements

Li-S batteries (CR 2032) are assembled in a glovebox filled with inert gas (H_2_O, O_2_ < 0.1 ppm). Lithium foil as the anode, S/C composites as cathode, and 1.0 M LiTFSI dissolved in a DOL/DME (volume ratio is 1:1) mixed solvent with 0.1 M LiNO_3_ additive as electrolyte. The amount of electrolyte used in each cell is 15 µl. For high sulfur loading of 3.1 mg cm^−2^, a low electrolyte/sulfur (E/S) ratio of ∼5 µl mg^−1^ was used.

### Materials Characterization and Electrochemical Analysis

The crystal phase, morphologies, and microstructure of the different samples were characterized with X-ray diffraction (XRD, Ultima IV, CuKα radiation), scanning electron microscope (SEM, Sigma500), and transmission electron microscope (TEM, JSM-2100 Plus), respectively. X-ray photoelectron spectrometer (XPS, PHI 5000) was used to survey the composition of elements and chemical state. Thermogravimetric analysis (TGA, TG 209 F3) was used to estimate the S content of the S/KB cathode. Electrochemical workstation (CHI 760E) was used to measure the original date of cyclic voltammetry (CV, the voltage range is 1.7–2.8 V with 0.1 mV S^−1^ scan rate) and electrochemical impedance spectroscopy (EIS, the AC voltage amplitude is 5 mV and the frequency range is 0.01 Hz–100 kHz). The electrochemical performance and constant current charge/discharge curve were gauged by Land CT 2001A battery test system in the 1.7–2.8 V voltage range.

## Results and Discussion

**FIGURE 1:**
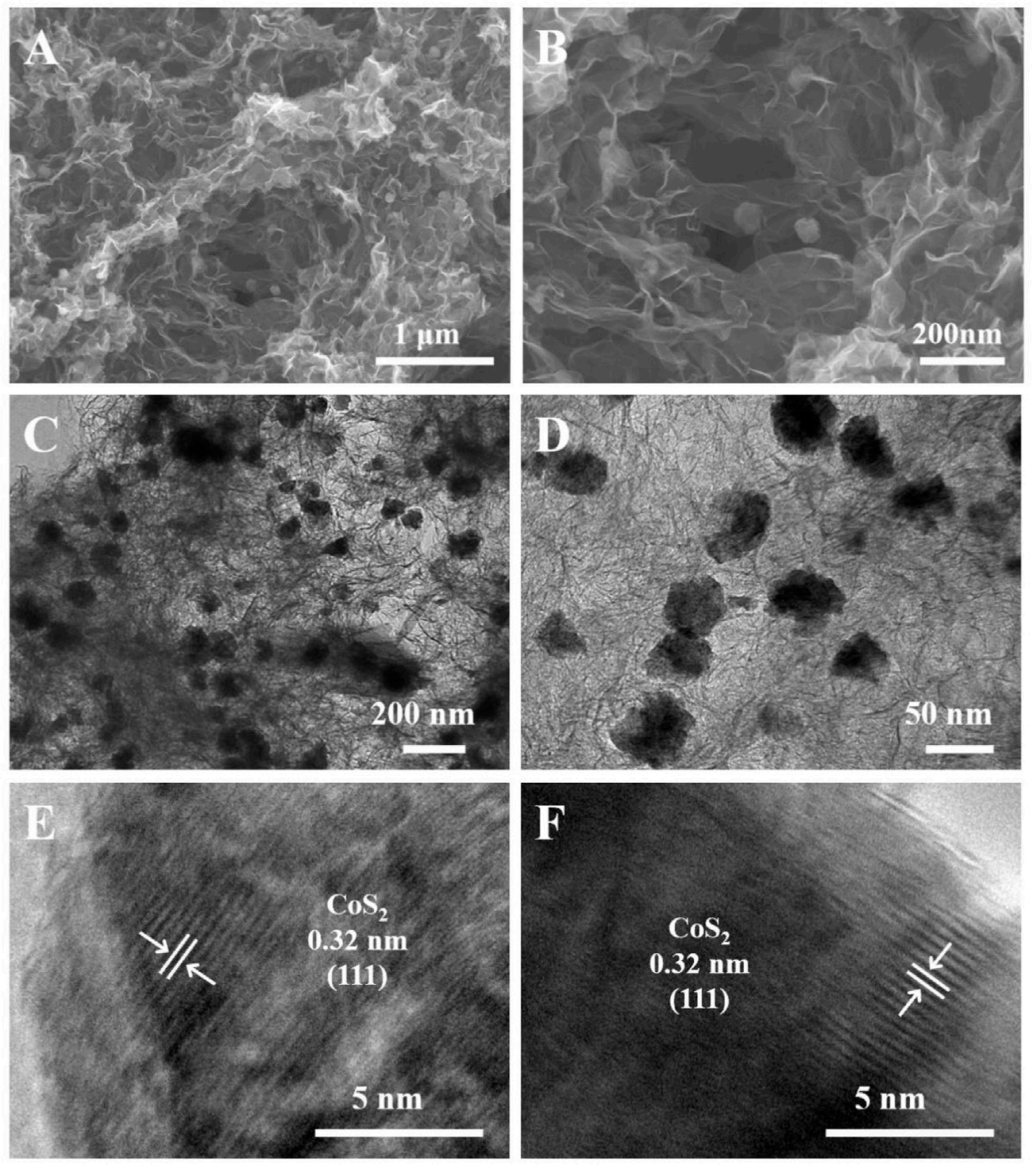
**(A,B)** SEM, **(C,D)** TEM, and **(E,F)** HRTEM images of the CoS_2_/rGO.

Figures 1A, B show the morphologies and microstructure of rGO and CoS_2_/rGO composites investigated by SEM; CoS_2_/rGO composites present a highly 3D porous structure, ensuring enough space to store LiPSs and accelerate ion diffusion (Zhao M. et al., 2020). CoS_2_ particles were evenly distributed on rGO sheet without any agglomeration, which is conducive to chemical adsorption of LiPSs. Supplementary Figures S1A,B show the rGO has a similar porous structure to CoS_2_/rGO composites. The high conductivity porous rGO framework forms the second current collector and ensures the rapid supply of electrons for electrochemical reactions (Chong et al., 2018). The TEM images of the CoS_2_/rGO composites in Figures 1C, D clearly show that the CoS_2_ particles with a diameter of about 50 nm were uniformly attached to the rGO sheet, matching well with the SEM images results. The high-resolution TEM (HRTEM) images in Figures 1E, F exhibit that the parallel lattice fringe is 0.32 nm, which can be indexed to the (111) crystal plane of CoS_2_ particle.

**FIGURE 2:**
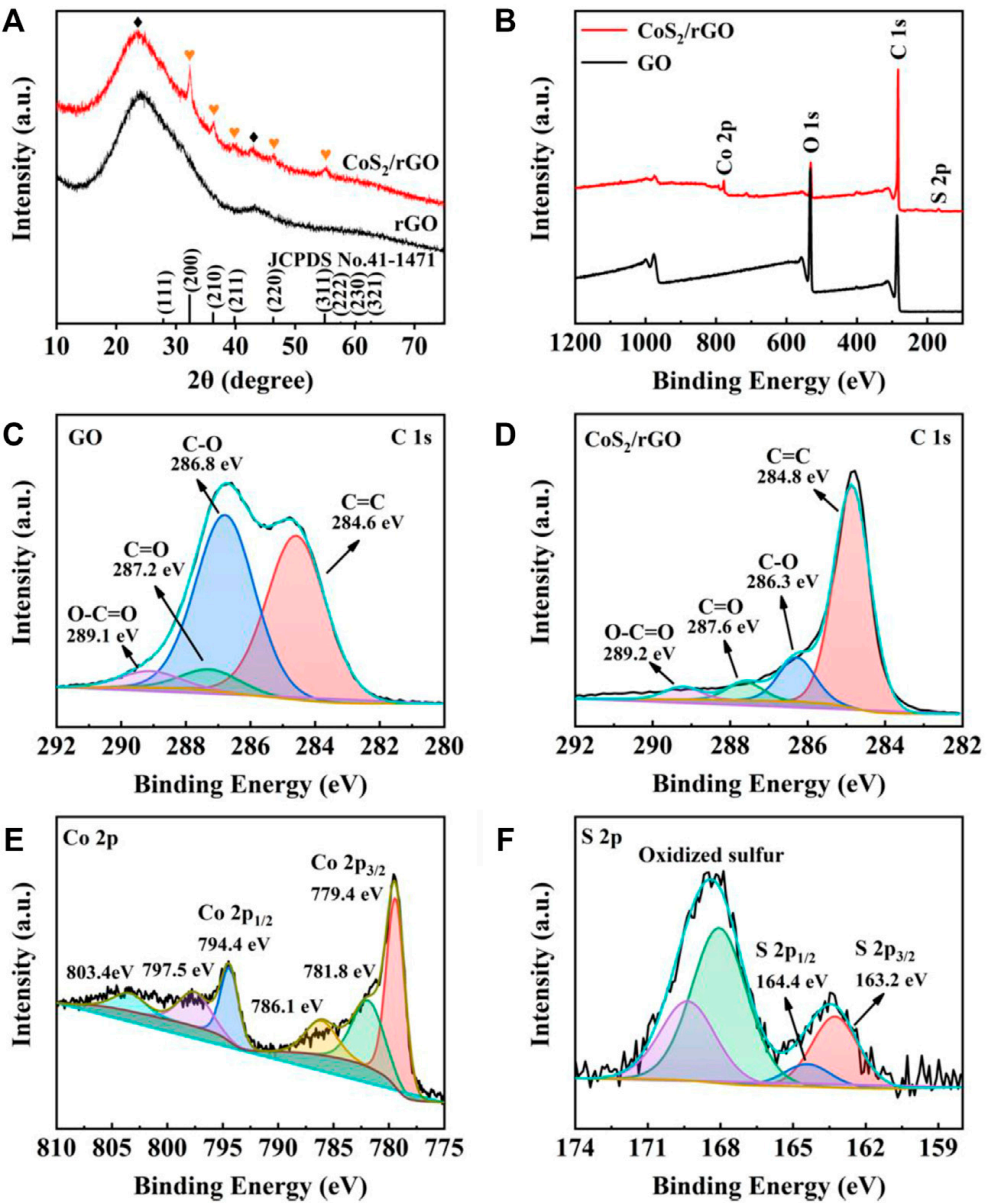
**(A)** XRD patterns of rGO and CoS_2_/rGO; **(B)** full XPS spectrum of GO and CoS_2_/rGO; high-resolution XPS (HR-XPS) spectra of C 1s **(C)** for GO; C 1s **(D)**, Co 2p **(E)**, and S 2p **(F)** for CoS_2_/rGO.

The phase of CoS_2_/rGO was studied *via* XRD, as shown in Figure 2A; the sharp diffraction peaks in the CoS_2_/rGO matched well with the characteristic peak of cubic CoS_2_ (PDF#41-1,471). No other impurity phases exist except for the characteristic peak of rGO at ∼25°. For confirming the chemical valence and element of GO and CoS_2_/rGO composites, XPS test result is shown in Figure 2B; the fitting curves present the presence of Co, S, and C components in CoS_2_/rGO materials, consistent with the XRD results. In particular, Figure 2C exhibits the C 1s HR-XPS spectrum of GO, four peaks at 289.1, 287.2, 286.8, and 284.6 eV, representing O-C=O, C=O, C-O, and C=C, respectively (Yuan, et al., 2015). As expected, the peak intensity ratio of these oxygen-containing functional groups in the CoS_2_/rGO is much lower than the peak intensity ratio in the GO sample (Figure 2D); this comparison demonstrated that GO can be effectively reduced to rGO during the microwave hydrothermal process. As shown in Figure 2E, the Co 2p_1/2_ and Co 2p_3/2_ characteristic peaks of CoS_2_ were located at 794.4 and 779.4 eV in the HR-XPS of Co 2p, respectively. (Xu, et al., 2021). The satellite peak signal is 781.8, 786.1, 797.5, and 803.4 eV (Wang, et al., 2018). The fitting results of Figure 2F for the HR-XPS of S 2p of the characteristic peak of S 2p_1/2_ and S 2p_3/2_ are shown at 164.4 and 163.2 eV, which corresponds to S 2p_1/2_ and S 2p_3/2_ of CoS_2_. The HR-XPS of S 2p detected peaks at 168.1 and 169.3 eV indicating the presence of sulfur oxides (Liu et al., 2020c). Therefore, the results demonstrate the successful preparation of CoS_2_/rGO material by the microwave hydrothermal method.

**FIGURE 3:**
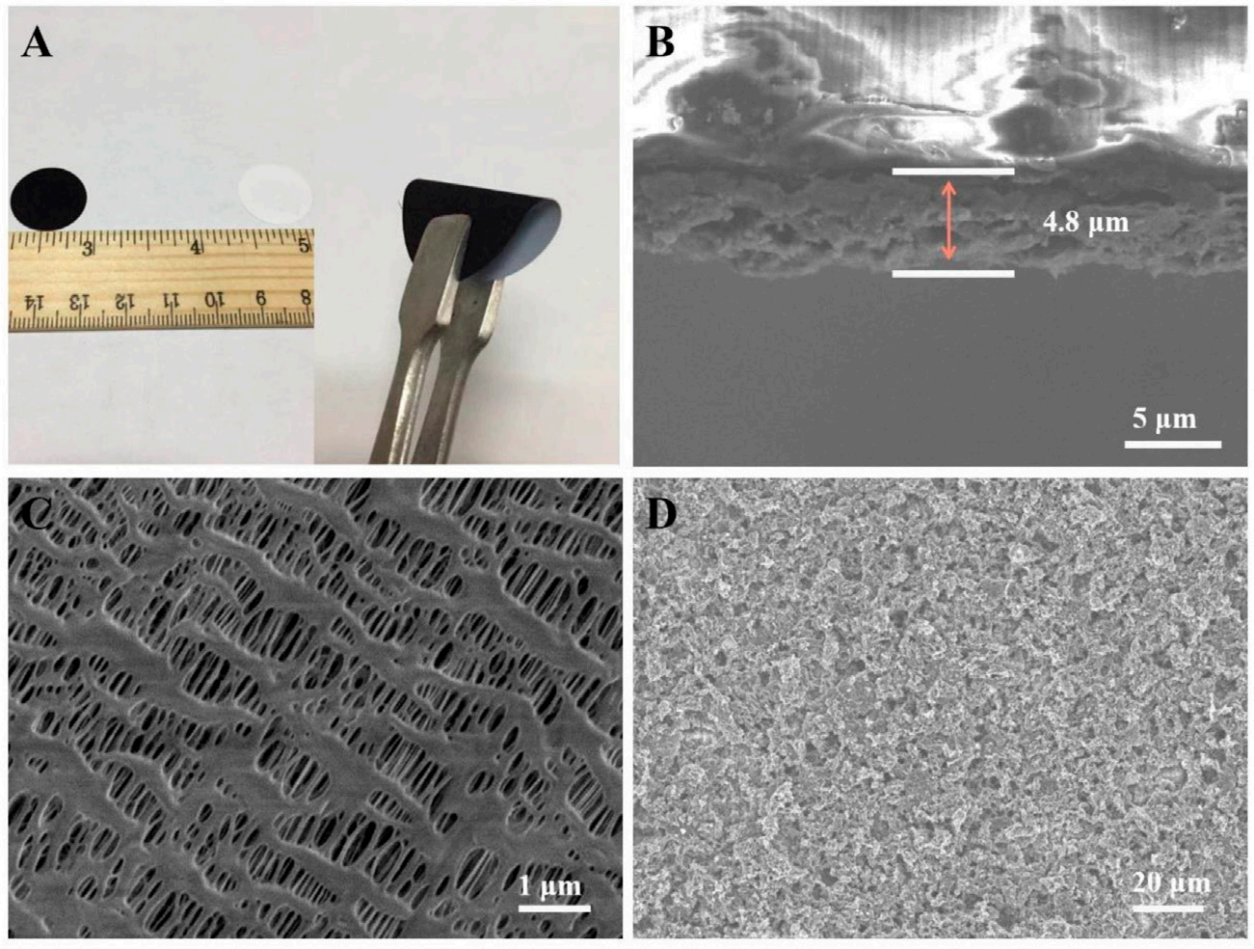
**(A)** Digital images of the CoS_2_/rGO modified separator; **(B)** cross-sectional SEM image of the CoS_2_/rGO modified separator; **(C,D)** top-view SEM image of the PP separator and CoS_2_/rGO modified separator, respectively.

Figure 3A shows a uniform coating surface and good mechanical flexibility of the CoS_2_/rGO modified separator. The cross-sectional image indicates that the coating thickness of CoS_2_/rGO functional interlayer is about 4.8 µm (Figure 3B). Figure 3C shows the top view SEM morphology of PP separator, and it can be seen that the PP separator possesses abundant pores with a size of 100–200 nm in width and micron scale in length, which is conducive to the rapid transmission of ions in electrolyte, while inevitably allowing the shuttle of soluble LiPSs in the pores, resulting in the existence of side reactions and irreversible loss of sulfur active substances. (Balach, et al., 2015; Xiong, et al., 2021). However, the top view SEM image of CoS_2_/rGO modified separator in Figure 3D indicates that the CoS_2_/rGO functional interlayer is evenly covered on the surface of PP separator to form a compact physical layer to block the migration of LiPSs and retains abundant pores to ensure fast ion transfer, which is similar with the surface of rGO layer (Supplementary Figure S2B).

**FIGURE 4:**
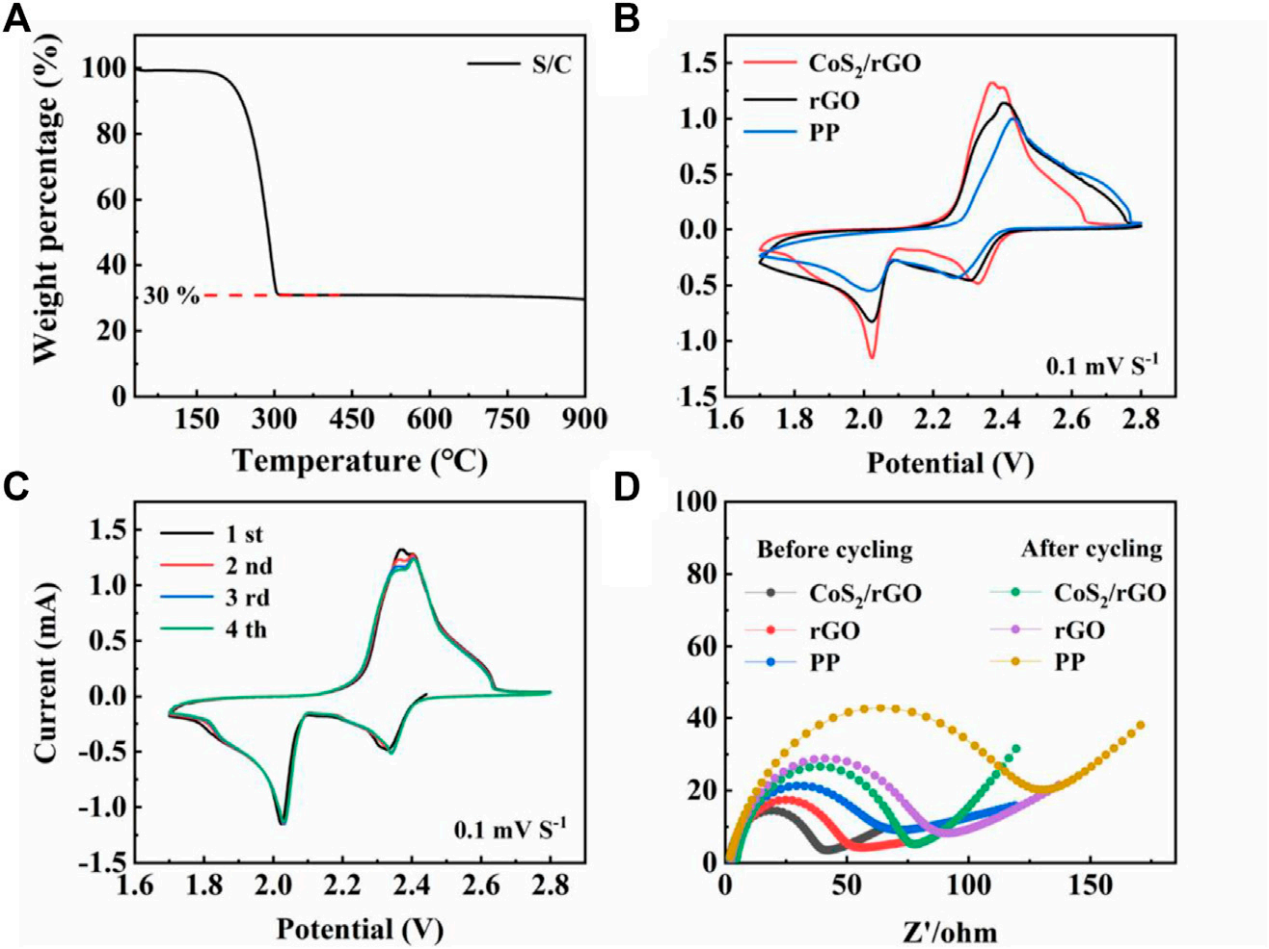
**(A)** TGA curves of S/C; **(B)** CV curves of the Li-S cells with different separators under 0.1 mV S^−1^; **(C)** CV curves of Li-S cell with CoS_2_/rGO modified separator for four cycles at the scan rate of 0.1 mV S^−1^; **(D)** Nyquist plots of Li-S cells with different separators before and after cycling.

The Li-S batteries with PP separator, rGO, and CoS_2_/rGO modified separators were subjected to the corresponding electrochemical tests for proving the superiority of CoS_2_/rGO modified separator in electrochemical performance. The S/C compound is used as the cathode electrode. TGA confirms the content of S in the S/C precursor is 70% (Figure 4A). Figure 4B shows the CV curves of the Li-S batteries with a PP separator, rGO modified separator, and CoS_2_/rGO modified separator between the voltages of 1.7 and 2.8 V at the scanning rate of 0.1 mV S^−1^. Two typical cathodic peaks were located at 2.33 and 2.02 V, which corresponds to the reduction process from solid S_8_ to soluble LiPSs and then further to solid-phase Li_2_S_2_/Li_2_S (Fan et al., 2019), while the continuous anodic peak at 2.36–2.40 V was attributed to the reversible oxidation of sulfur species to S_8_ (Zhuang et al., 2020). It can be seen from Figure 4B that, compared with PP separator and rGO modified separator, the Li-S battery with CoS_2_/rGO modified separator shows sharper redox peaks and higher peak current responses, which can be owing to the robust interaction for LiPSs and the accelerated redox kinetics by the electrochemical catalytic ability of CoS_2_ (Li et al., 2021; Yuan et al., 2017). At the same time, Figure 4C shows the CV curves of a Li-S battery with a CoS_2_/rGO modified separator for four cycles. The well-overlapped curves with each other further proved the good reversibility and the strong immobilization ability of LiPSs (Liu, et al., 2019). EIS of the Li-S batteries with PP separator, rGO, and CoS_2_/rGO modified separators before cycling and after 50 cycles was conducted and shown in Figure 4D; the diameter of the semicircle in the low-frequency region represents the charge transfer resistance (R_ct_) (Deng et al., 2013; Cui et al., 2021). It can be seen that fresh cells with CoS_2_/rGO modified separator exhibited the smallest R_ct_ value when compared with PP separator and rGO modified separator, which indicates that CoS_2_ uniformly attached in rGO could effectively ensure fast transmission for Li^+^ migration and reduce the charge transfer resistance. After cycling at 1 C for 50 cycles, the values of R_ct_ show an increase, which can be ascribed to the redistribution of S and the accumulation of Li_2_S and Li_2_S_2_ in porous structure. Li-S battery with CoS_2_/rGO modified separator still shows the smallest R_ct_ value. change than that of the other two cells after cycling, suggesting that CoS_2_/rGO modified separator could effectively ease the accumulation of LiPSs and improve the utilization of sulfur active materials (Liu et al., 2020a; Ma et al., 2021b).

**FIGURE 5:**
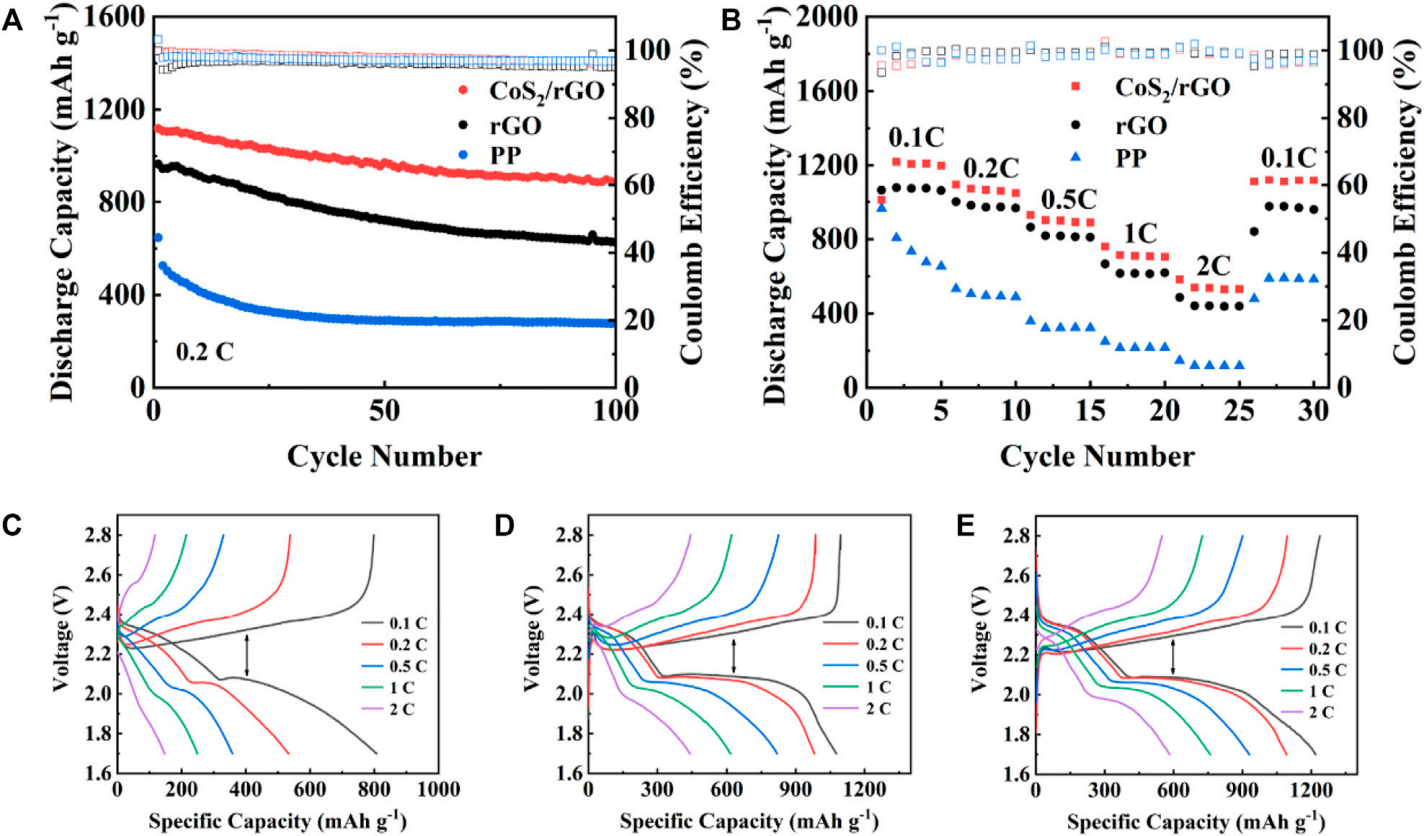
**(A,B)** Cycling and rate performance of the Li-S batteries with CoS_2_/rGO, rGO, and PP separator at 0.2 C; discharge/charge profiles of cells with PP **(C)**, rGO **(D)**, and CoS_2_/rGO **(E)** separators at different rates.

Figure 5A appraises the cycle stability of the Li-S batteries with different separators for 100 cycles at 0.2 C. The cells with CoS_2_/rGO modified separator show the highest first discharge capacity of 1,122.3 mAh g^−1^. It is worth noting that the specific capacity remains at 897.8 mAh g^−1^ with a capacity retention rate of 80% after 100 cycles. In contrast, the initial discharge capacity of the batteries with rGO and PP separator are 970.7 and 646.9 mAh g^−1^ at 0.2 C; after 100 cycles, the capacity only remains at 631.2 and 275.9 mAh g^−1^, respectively (the capacity retention rates are 65% and 42%). In addition, the Li-S battery with CoS_2_/rGO modified separator also exhibits excellent rate performance. As shown in Figure 5B. The discharge capacity of Li-S battery with CoS_2_/rGO modified separator at 0.1–1 C is 1,218.3, 1,093, 930.2, and 760.1 mAh g^−1^, respectively. Even at a high current density of 2 C, the corresponding capacity is still 583.9 mAh g^−1^. When the current density is converted back to 0.1 C, the reversible discharge capacity is 1,110.5 mAh g^−1^, indicating its high reversibility. Furthermore, the charge/discharge profiles of batteries with different separators at 0.1–2 C current density are also investigated; the Li-S battery with CoS_2_/rGO modified separator shows a more stable and flatter potential platform than Li-S batteries and other separators at different current densities (Figure 5C) at different current rates of charge and discharge, and the platform reflects its better electrochemical accessibility. Besides, the degree of electrochemical polarization gradually intensifies with the increases of current density; as shown in Figures 5C–E, the Li-S battery using CoS_2_/rGO modified separator shows the lowest potential plateau gaps between different charge/discharge profiles than others, with platform potential difference of 168, 187, 248, 303, and 410 mV at 0.1–2 C, suggesting the excellent electrocatalysis of polar CoS_2_ nanoparticles could alleviate electrochemical polarization. The above results demonstrate that the CoS_2_/rGO modified separator could effectively capture LiPSs and catalyze its conversion to Li_2_S_2_/Li_2_S, thus achieving fast redox reaction kinetics and suppressed shuttle effect.

**FIGURE 6:**
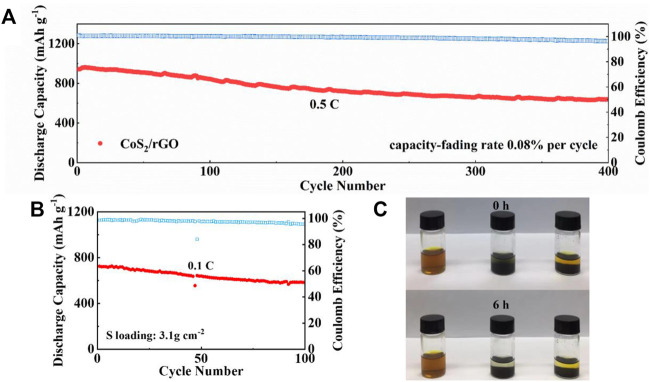
**(A)** The long cycling performance of the Li-S battery with CoS_2_/rGO separator at 0.5 C for 400 cycles; **(B)** cycle stability of the Li-S battery with CoS_2_/rGO separator of high S loading at 0.1 C; **(C)** digital images of Li_2_S_6_ adsorption test for 0 and 6 h, respectively.

The long-term cyclabilities of the Li-S battery with CoS_2_/rGO modified separator were further evaluated in Figure 6A. At a discharge current density of 0.5 C, the first discharge capacity of the Li-S battery with the CoS_2_/rGO modified separator is 963.2 mAh g^−1^. After 400 cycles, the discharge capacity remains at 640.8 mAh g^−1^, and the average capacity decay rate per cycle is only 0.08%; meanwhile, the average Coulombic efficiency of 98.3% was achieved. The stable Coulombic efficiency indicates that the CoS_2_/rGO interlayer effectively inhibits the shuttle effect and the irreversible wastage of lithium metal. More satisfyingly, after four cycles activated at 0.02 C, the battery can deliver reliable stability under high S loading of 3.1 mg cm^−2^; the capacity reaches 725.1 mAh g^−1^ at 0.1 C and remains at 583.4 mAh g^−1^ after 100 cycles with a high capacity retention ratio of about 80% (Figure 6B). This shows that the Li-S battery with CoS_2_/rGO modified separator has great potential for practical applications. Meanwhile, a plain visual Li_2_S_6_ adsorption test was used to measure the capability of the CoS_2_/rGO to capture LiPSs. With the same mass of CoS_2_/rGO and rGO immersed in equal volume brown Li_2_S_6_ solution, in Figure 6C, after static holding for 6 h, the color of the solution in the bottle containing rGO retains pale yellow, indicating that rGO only has poor physical adsorption to LiPSs. In sharp contrast, the color of the solution for CoS_2_/rGO changed from brown to nearly transparent, which clearly confirms the effective chemical interaction of CoS_2_/rGO with LiPSs.

## Conclusion

In summary, we report a simple and energy-saving synthesis of CoS_2_/rGO composites *via* a facile microwave hydrothermal method and applied it as an efficient mediator to trap LiPSs and accelerate its reaction kinetics to reinforce the electrochemical performance of Li-S battery. In this structure, high conductivity rGO network can shorten the path of ion migration and therefore reduce the internal resistance of cells. The porous structure of rGO combining the polar sites of CoS_2_ nanoparticles could fix the soluble LiPSs on the cathode side through physical and chemical adsorption and enhance redox kinetics as an efficient electrochemical catalyst, thereby efficiently alleviating the shuttle effect of the LiPSs through the separator. Therefore, the Li-S battery with CoS_2_/rGO modified separator exhibits excellent rate performance and stable cycling performance under high sulfur loading of 3.1 mg cm^−2^.

## Data Availability

The raw data supporting the conclusion of this article will be made available by the authors, without undue reservation.
