# Supplementary material for: Energy-Saving Synthesis of Functional CoS2/rGO Interlayer With Enhanced Conversion Kinetics for High-Performance Lithium-Sulfur Batteries
Source: Front Chem. 2022 Feb 10;9:830485. doi: 10.3389/fchem.2021.830485 (PMC8867214; doi:10.3389/fchem.2021.830485)
Supplement: Supplementary file 1 [file DataSheet1.docx]

**Supporting information**

**Energy-Saving Synthesis of Functional CoS_2_/rGO Interlayer with Enhanced Conversion Kinetics for High-Performance Lithium-Sulfur Batteries**

Junan Feng^1†^, Yahui Li^1†^, Jinshi Yuan^1^, Yuling Zhao^1^, Jianmin Zhang^2^, Fengyun Wang^1^, Jie Tang^3^*, Jianjun Song^1^*

^1^ College of Physics, Qingdao University, Qingdao 266071, China

^2^ National Engineering Research Center for Intelligent Electrical Vehicle Power System (Qingdao), College of Mechanical and Electrical Engineering, Qingdao University, Qingdao 266071, China

^3^ National Institute for Materials Science, Tsukuba 305-0047, Japan

***Corresponding Author:**

*Email addresses:* tang.jie@nims.go.jp (J. Tang); Jianjun.song@qdu.edu.cn (J. Song).

^†^ These authors have contributed equally to this work.


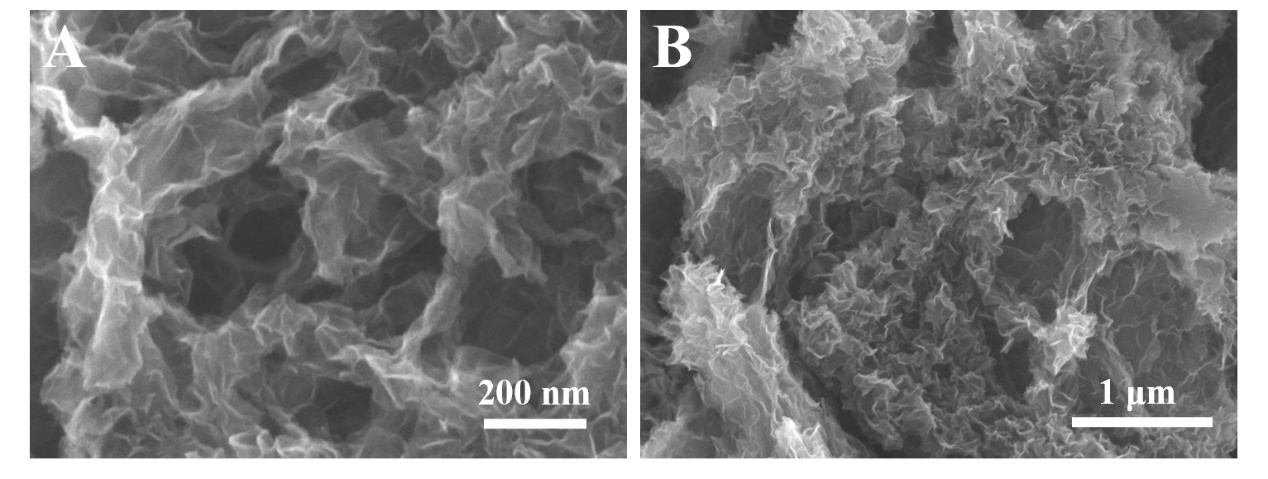


Figure S1 The SEM images of rGO.


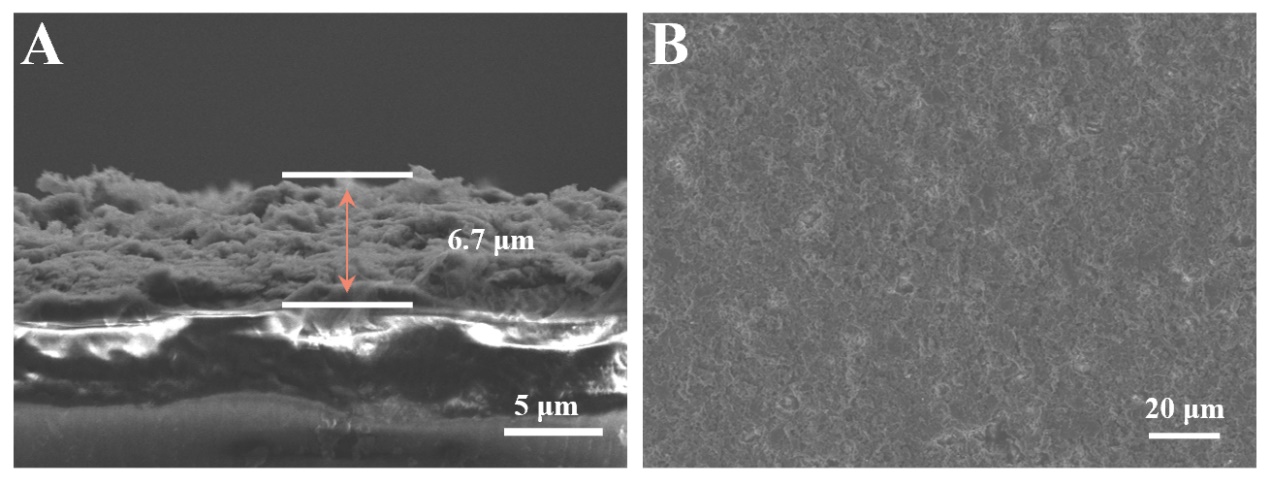


Figure S2 (A) The cross-sectional and (B) top view SEM images of rGO modified separator
